# Epidermal T Cell Dendrites Serve as Conduits for Bidirectional Trafficking of Granular Cargo

**DOI:** 10.3389/fimmu.2018.01430

**Published:** 2018-06-22

**Authors:** Grzegorz Chodaczek, Monika Toporkiewicz, M. Anna Zal, Tomasz Zal

**Affiliations:** ^1^Department of Immunology, University of Texas MD Anderson Cancer Center, Houston, TX, United States; ^2^Confocal Microscopy Laboratory, Wroclaw Research Centre EIT+, Wroclaw, Poland

**Keywords:** gamma delta (gammadelta) T cells, dendritic epidermal T cell, lysosomes, intracellular transport, intravital microscopy, fluorescence recovery after photobleaching

## Abstract

Dendritic epidermal T cells (DETCs) represent a prototypical lineage of intraepithelial γδ T cells that participate in the maintenance of body barrier homeostasis. Unlike classical T cells, DETCs do not recirculate and they remain persistently activated through their T cell receptors (TCR) at steady state, i.e., in absence of infection or tissue wounding. The steady state TCR signals sustain the formation of immunological synapse-like phosphotyrosine-rich aggregates located on projections (PALPs) which act to anchor and polarize DETC’s long cellular projections toward the apical epidermis while the cell bodies reside in the basal layers. The PALPs are known to contain pre-synaptic accumulations of TCR-containing and lysosomal granules, but how this cargo accumulates there remains unclear. Here, we combined anti-Vγ5 TCR, cholera toxin subunit B (CTB), and LysoTracker (LT)-based intravital labeling of intracellular granules, with high resolution dynamic microscopy and fluorescence recovery after photobleaching (FRAP) to characterize the steady state composition and transport of DETC granules in steady state epidermis. Intradermal fluorescent Vγ5 antibody decorated DETCs without causing cellular depletion, dendrite mobilization or rounding up and became slowly internalized over 48 h into intracellular granules that, after 6 days, colocalized with LAMP-1 and less so with LT or early endosomal antigen-1. Intradermal CTB was likewise internalized predominantly by DETCs in epidermis, labeling a partly overlapping set of largely LAMP-1^+^ intracellular granules. These as well as LT-labeled granules readily moved into newly forming dendrites and accumulated at the apical endings. FRAP and spatiotemporal tracking showed that the inside tubular lengths of DETC cellular projections supported dynamic trafficking of lysosomal cargo toward and away from the PALPs, including internalized TCR and lipid raft component ganglioside GM1 (labeled with CTB). By contrast, the rate of GM1 granules transport through comparable dendrites of non-DETCs was twice slower. Our observations suggest that DETCs use chronic TCR activation to establish a polarized conduit system for long-range trans-epithelial transport aimed to accumulate mature lysosomes at the barrier-forming apical epidermis. The biological strategy behind the steady state lysosome polarization by DETCs remains to be uncovered.

## Introduction

Considered a member of the innate body barrier defense system, murine dendritic epidermal T cells (DETCs) contribute to skin repair and homeostasis (1–3). These cells extend long cellular processes from the mid-body in the basal epidermis toward the apical epidermis thereby spanning across the whole epidermis thickness and interacting with both the immature (basal) and mature (squamous) keratinocytes. The striking apical polarity of DETCs results from the formation of dendrite-anchoring phosphotyrosine-rich aggregates located on projections (PALPs) which are sustained at inter-squamous keratinocyte junctions by the local chronic activation of the cell’s unique Vγ5-Vδ1 T cell receptors (TCR) [according to the Tonegawa nomenclature (4, 5)]. The dendrite-terminal cytoplasm underneath the PALPs harbors distinct accumulations of intracellular granules some of which contain TCR and/or lysosomal and exocytic pathway markers LysoTracker (LT), GM1, and LAMP-1 (5). The presence of intracellular granules inside DETCs was uncovered as early as in 1985 by Romani et al. who described electron-dense cores and small vesicles surrounded by less electron-dense material in isolated Thy-1^+^ epidermal cells (DETC) (6). Thereafter, and consistent with the capacity of DETCs to kill their targets, Krähenbühl et al. demonstrated that some DETC granules contained granzyme A (BLT esterase activity) and perforin (Ca^2+^-dependent hemolytic activity) (7) and Ibusuki et al. demonstrated *in vitro* cytotoxic granule exocytosis in response to stimulation of short-term DETC lines (8). The steady state accumulations of dendrite-terminal granular cargo at the PALPs could signify a local formative process, such as TCR and/or other membrane component internalization and/or a long-range transport from the cell bodies, and the cargo could be poised for localized secretion. However, the *in vivo* behavior of DETC’s intracellular cargo has remained unknown.

In this work, we establish a methodology for the labeling and DETC-selective analysis of intracellular cargo transport *in vivo*, and we apply it to examine the dynamic behavior of DETC’s intracellular granules at steady state conditions. Using time-lapse fluorescence microscopy and fluorescence recovery after photobleaching (FRAP), we show that DETC TCR and GM1 membrane components are readily internalized and that the content of resulting cargo enters the lysosomal and LAMP-1 granule pools. Furthermore, we show that DETCs transport their intracellular granules along the lengths of the cell’s a dendrites, at steady state, both away from and toward the apical epidermis, and more dynamically than similarly labeled dendritic-form dermal cells. Our observations demonstrate a novel approach to study DETC cargo dynamics and suggest that DETCs use chronic, dendrite-terminal TCR activation to establish a polarized conduit system for trans-epithelial cargo transport.

## Materials and Methods

### Mice

IL2p8-GFP mice were obtained from M. Yui and E. Rothenberg (California Institute of Technology, Pasadena, CA, USA) (9) and used at 6–24 weeks of age. In these mice, the epidermal GFP is present solely in DETCs (5, 9). CD11c-YFP mice (10) were obtained from M. Nussenzweig (The Rockefeller University, New York, NY, USA) and were crossed with IL2p8-GFP mice to simultaneously visualize DETCs and Langerhans cells. The mice were housed at the University of Texas MD Anderson Cancer Center (UT MDACC), Houston, TX, USA and the Wroclaw Research Centre EIT+ and the Institute of Immunology and Experimental Therapy, Wroclaw, Poland in individually ventilated cages in 12:12 h light-dark cycle under specific pathogen-free conditions. All animal manipulations were approved by the UT MDACC Institutional Animal Care and Use Committee or the Local Ethics Committee for Experiments on Animals at the Institute of Immunology and Experimental Therapy.

### Antibodies and Reagents

LysoTracker Red DND-99, cholera toxin subunit B (CTB)-Alexa Fluor (AF)555 or AF647 conjugates (Cat. No A20187, A10470, and A20186), and anti-rabbit IgG-AF647 antibody were from ThermoFisher Scientific. Unlabeled anti-TCR Vγ3 (Vγ5 according to Tonegawa’s nomenclature) antibody (clone 536) was from Santa Cruz Biotechnology and its isotype control (Syrian hamster IgG, clone SHG-1) was from BioLegend. These antibodies were labeled in house using AF555 and AF647 labeling kits according to manufacturer’s instructions (ThermoFisher Scientific). Anti-LAMP-1-AF647 clone 1D4B was from BioLegend and anti-early endosome antigen-1 (EEA-1) (#2411) was from Cell Signaling.

### *Ex Vivo* Immunofluorescence of Mouse Epidermis

Mouse ears were split laterally and immediately fixed for 1 h at 20–22°C with 3.7% (wt/vol) formaldehyde. The subcutaneous cartilage was removed and the skin was made permeable for at least 18 h with 0.5% (wt/vol) saponin in 2% (vol/vol) FBS and 0.03% (wt/vol) azide in PBS. Samples were stained for at least 18 h at 22–37°C with antibodies diluted in 2% (vol/vol) FBS and 0.5% (vol/vol) saponin in PBS and, after being washed in PBS, were mounted in ProLong Gold (ThermoFisher Scientific). Fluorescence imaging was performed using a Leica SP8 confocal microscope with 63× NA1.4 and 40× NA1.3 oil objectives (Leica Microsystems).

### Intravital Labeling and Microscopy

Mice were anesthetized and the ear pinnae was injected, using 31 G insulin syringe, with 20 µl of the following labeling solutions, in PBS: 10 µM LT Red, 10 µg/ml CTB-AF555 or AF647 conjugate, ~1.4 μg/ml fluorescently labeled anti-Vγ5 antibody, or ~1.4 μg/ml of fluorescently labeled isotype control antibody. In some experiments, CTB and anti-Vγ5 antibody were mixed together. Intravital imaging was performed 1 h later for LT, or several days later for the antibodies. Mice were anesthetized by isoflurane inhalation and placed on a heated microscope stage. Ear pinna was immobilized on a metal pedestal with a dab of silicone paste, moistened with a drop of PBS and covered with a 0.17-mm glass coverslip. The imaging was performed using upright Leica SP5 or SP8 resonant scanning confocal systems equipped with piezoelectric z-drive (Piezosystem, Jena) and 40× NA1.3 oil objective. The pinhole was set to 1–2 AU and image pixel size at 0.09–0.150 µm. Stacks of confocal images, spaced 0.1–1 µm apart, were acquired every 10–30 s for up to 2 h with line averaging to diminish noise.

### Image Processing

3-D and temporal image stacks were smoothed by kernel 3 median filtering and autofluorescence background was removed by thresholding followed by contrast stretching. Tissue drift was corrected using Imaris software (Bitplane) in 3-D or Stackreg plugin (11) of Fiji software (National Institutes of Health) in 2-D maximum intensity projections. For granule tracking, the GFP signal of DETCs was binarized and used to gate other fluorescence channels. Granule surfaces were identified and tracked in 2-D and shape parameters were measured using the Imaris software (Bitplane). The automated tracking algorithm was the autoregressive motion with 3 µm maximum one frame travel distance. Alternatively, granules were tracked manually. Two-dimensional projections of 3-D z-stacks were generated based on maximum intensities. Depth was color-coded using Leica Application Suite Advanced Fluorescence software or Temporal Color Code plugin in Fiji with “Rainbow RGB” look-up table. Co-localization analysis and Pearson coefficient calculations were performed in Imaris. All fluorescence intensity measurements in regions of interest (ROI) and line profiling were done using Fiji. To delineate mid-body regions, the GFP-based cell regions were eroded until dendrite disappearance followed by dilation. Dendrite regions were obtained by subtracting the perinuclear regions from the initial cell regions.

### Statistical Analysis

Statistical significance of differences between two experimental groups were determined using a nonparametric Mann–Whitney *U*-test (two-group comparison) with *p*-values of less than 0.05 considered significant, in GraphPad Prism (GraphPad Software).

## Results

### *In Vivo* Labeling of DETC Granules

**Figure 1:**
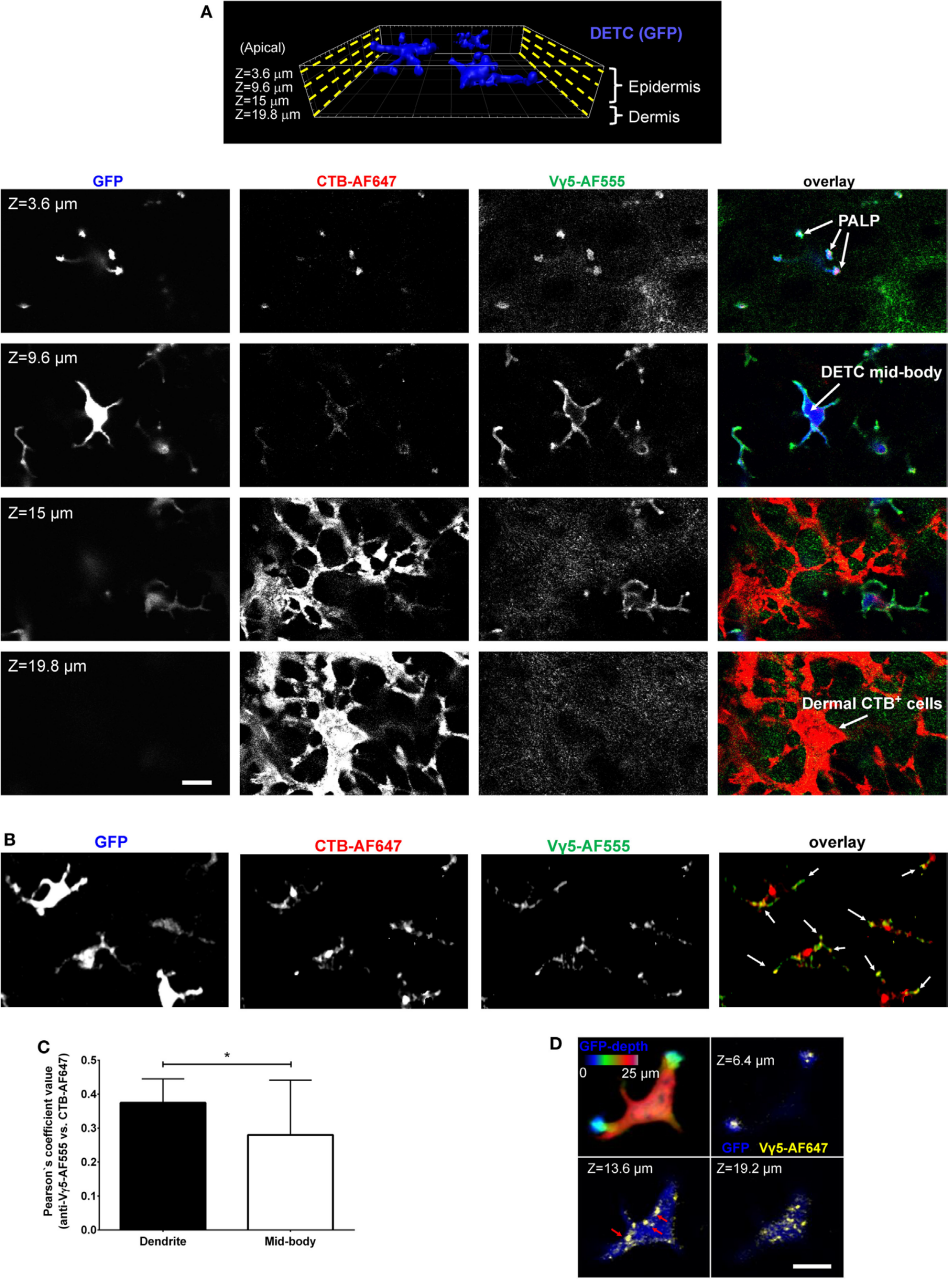
Intravital skin labeling by intradermal injection of fluorescent anti-Vγ5 antibody and CTB in IL2p8-GFP mouse ear. **(A)** One hour from the injection. Four representative confocal image z-planes from a 3-D z-stack at the indicated z-depth positions measured from the apical skin surface. **(B)** Six days after injection (different site). Maximum intensity projection of a confocal image z-stack. The white arrows point to the apical dendrite positions. **(C)** Quantification of Vγ5 and CTB signal co-localization after 6 days. **(D)** 3-D confocal sectioning to demonstrate the cytoplasmic localization of the granules 6 days after labeling. The color-coded depth projection shows the entire dendritic epidermal T cell (DETC) body. Scale bars = 10 µm.

In our previous report, we described the *in vivo* phenomenon of DETC steady state apical polarization through the dendrite-terminal TCR activation in the PALPs (5). Among other findings, we observed that besides in the PALPs membrane, TCR was present along with GM1 in juxtaposed cytoplasmic foci, and that much of DETC’s lysosomes (LT) and GM1 [cholera toxin subunit B (CTB) binding] were accumulating at steady state at the PALPs. We also noticed that intravital anti-CD3 cross-linking did not activate DETCs to round up. Therefore, to visualize TCR-containing DETC intracellular granules *in vivo*, we injected the dermis with AF647-labeled anti-Vγ5 TCR antibody [Tonegawa’s nomenclature (4)], also known as Vγ3 in Garman’s nomenclature (12). In addition, or separately, to label GM1-containing granules, we injected CTB-AF555. Both reagents diffused into the epidermis and stained preferentially DETCs within 10 min and delineated the plasma membranes with strong accumulations at the apical dendrite ends (Figure 1A; Figure S1 in Supplementary Material), thereby confirming *in vivo* the staining of the PALPs in fixed epidermis (5). We also noted that whereas the cellular specificity of Vγ5-AF647 and CTB was almost exclusive for DETCs in the epidermis (Figure 1, *Z* = 3.6–9.6 μm; Figure S2 in Supplementary Material), CTB also labeled a network of other, currently unidentified cells in the underlying dermis, many of them remarkably dendritic (Figure 1A, *Z* = 15–19.8 μm). CTB-AF555 became internalized into cytoplasmic granules within 90 min and Vγ5-AF647 was partially internalized after 24 h (Figure S1 in Supplementary Material) and completely after 6 days thereby highlighting a collection of intracellular granules (Figures 1B,D). The intravital labeling of DETC granules by Vγ5 antibody and CTB persisted for at least 16 days and we did not observe any loss of DETC cellularity or any signs of acute activation such as dendrite motility or cells rounding up.

Using intradermal Vγ5 and/or CTB fluorescence labeling, we focused further studies on the steady state, i.e., at least 6 days after the intradermal injection. Vγ5 fluorescence was found in ellipsoidal shaped granules measuring, in the XY image planes, respectively, 0.25 ± 0.007 µm × 0.48 ± 0.012 µm on the short and long axes (mean ± SEM). Given that the antibody could be proteolytically degraded over the time, the observed signals did not necessarily indicate a continued presence of TCR. Likewise, CTB-AF555 fluorescence delineated small granules dispersed throughout DETC body and prominent granular accumulations in the apical dendrite endings (Figure 1B; Figure S3 in Supplementary Material). These granules ellipsoid size was 0.27 ± 0.001 and 0.48 ± 0.001 µm (mean ± SEM). The Vγ5 and CTB fluorescence signals partly colocalized in a subset of intracellular granules, especially at the ends of dendrites (Figure 1C). The intracellular localization of the granules was ascertained by 3-D confocal sectioning (Figure 1D).

**Figure 2:**
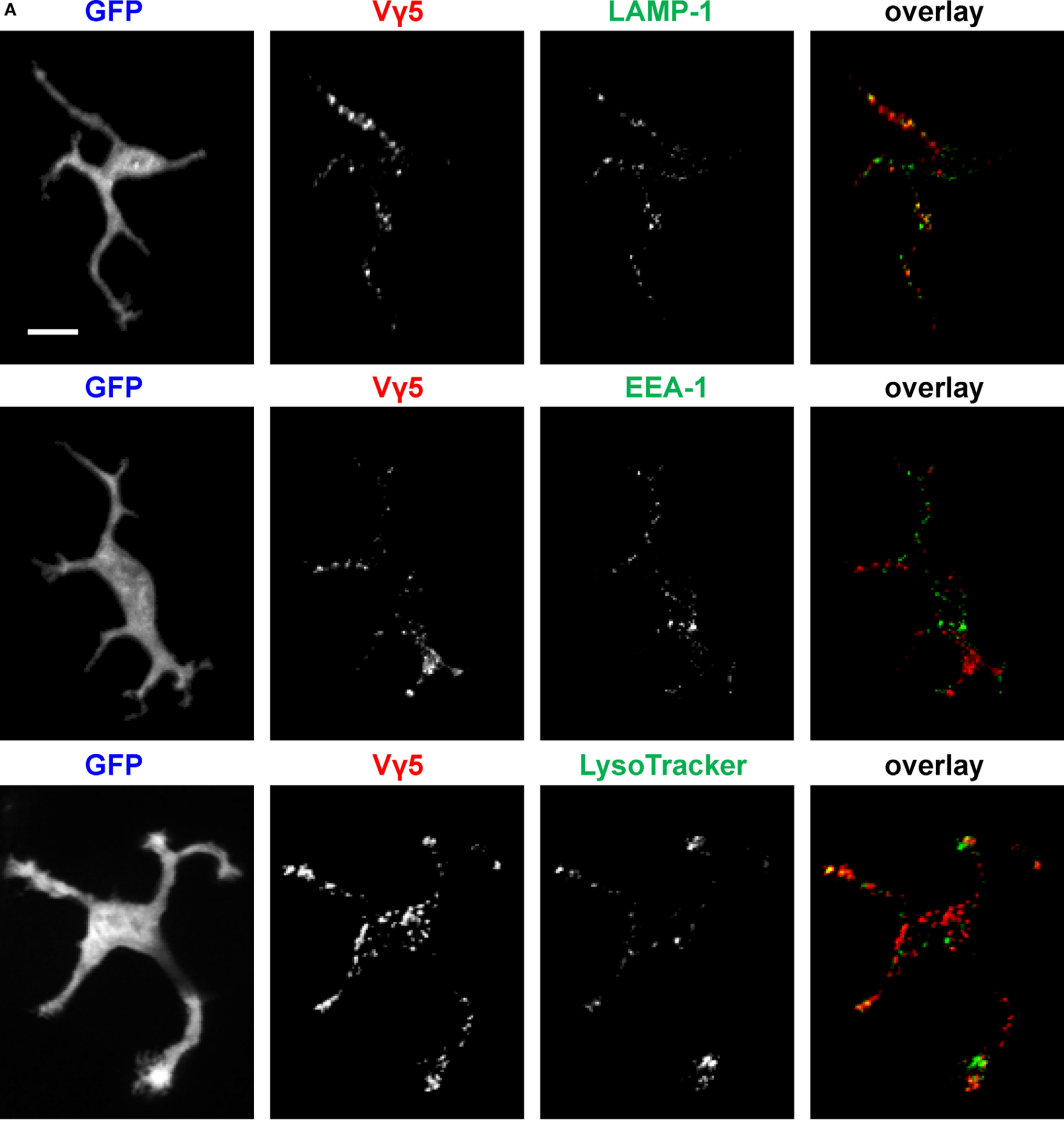

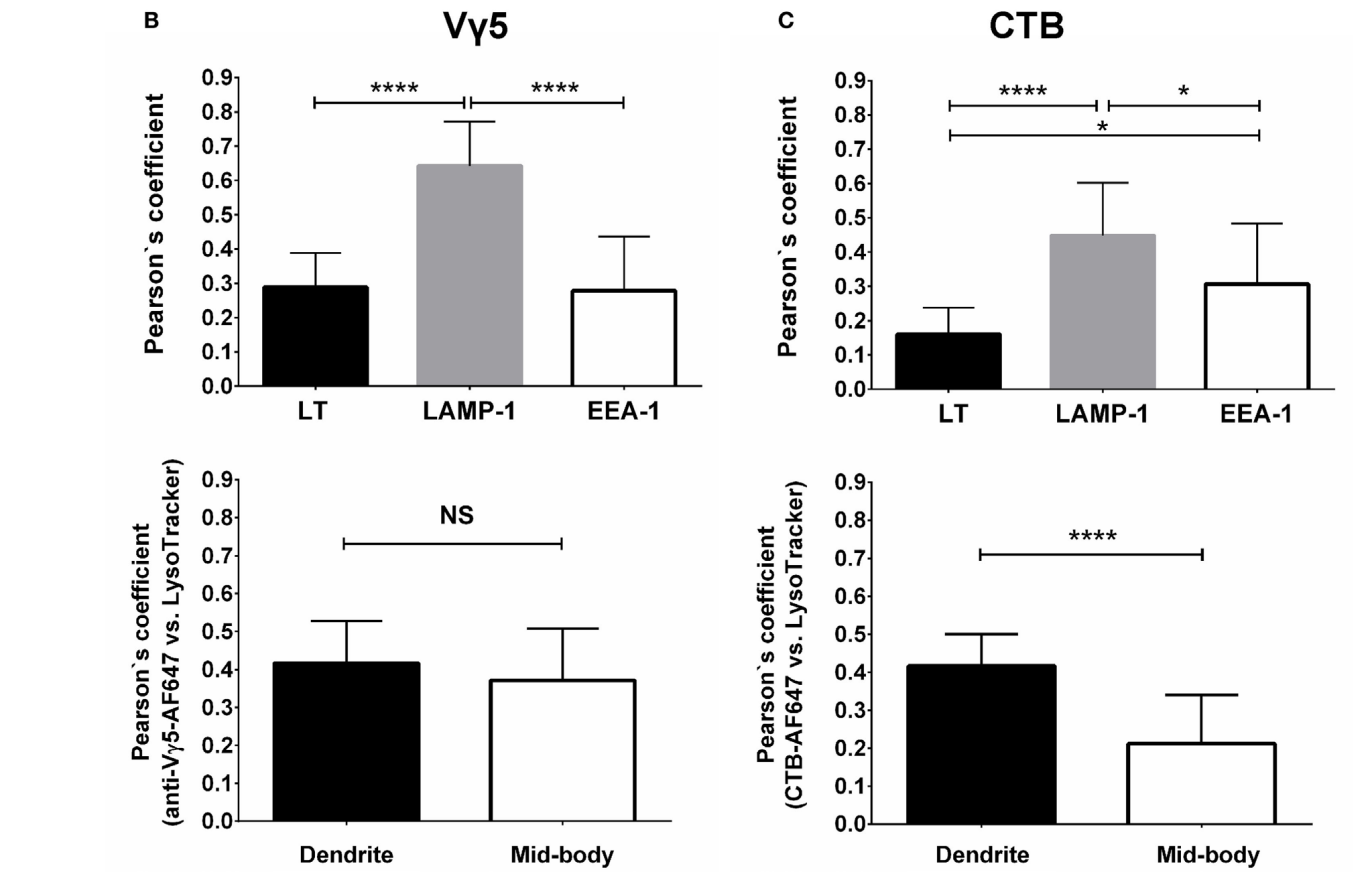
Characterization of dendritic epidermal T cell (DETC) granules containing internalized Vγ5 or CTB fluorescence. IL2p8-GFP mouse ear was injected with Vγ5-AF555 antibody or CTB-AF555 and, 6 days later, stained with LT or harvested for immunofluorescence. **(A)** Representative DETC examples. **(B)** Analysis of Vγ5^+^ granule co-localization with endosomal [early endosome antigen-1 (EEA-1)] and lysosomal [LAMP-1 and LysoTracker (LT)] markers in whole DETC bodies (upper panel) or in dendrites and mid-bodies (lower panel). **(C)** Similar as in **(B)**, but for CTB. *N* = 20–30 cells/mouse in five mice. The dendrite vs. mid-body co-localization analyses [**(B,C)** lower panels] were performed on a subset of the cells in upper panels (i.e., where mid-bodies could be clearly delineated). Scale bar = 10 µm.

To characterize the *in vivo* Vγ5 and CTB steady state DETC granules, we co-labeled the skin of IL2p8-GFP mice with intradermal LT *in vivo* followed by GFP-based image gating. We also stained the steady state Vγ5 and CTB-labeled epidermis *ex vivo* for the EEA-1 and LAMP-1 (Figure 2). In agreement with our prior report (5), LT-stained lysosomes were predominantly localized at the ends of dendrites. Within the limits of confocal resolution, LT granule’s diameters ranged from 0.25–0.45 ± 0.001 µm (mean ± SEM) to 0.8–1.0 µm [full width at half maximum (FWHM)]. Both Vγ5 and CTB signals most substantially colocalized with LAMP-1 and less so with EEA-1 and LT (Figures 2A–C; Figure S3 in Supplementary Material) and CTB/LT co-localization was significantly higher at dendrite ends than in mid-bodies (Figure 2C). Vγ5/LT co-localization in dendrites vs. mid-bodies was not significantly different, at the current statistical power. The Pearson’s coefficients in Figures 2B,C lower panels are not averages of the corresponding whole-cell values in the upper panels due to using a subset of cells with clearly defined mid-bodies, and ROI differences. Taken together, these results revealed a propensity of DETCs to internalize cell membrane TCR and GM1 into partially overlapping pools of lysosomal and exocytic pathway (LAMP-1) intracellular granules.

### Dynamics of LT-Stained Granules

**Figure 3:**
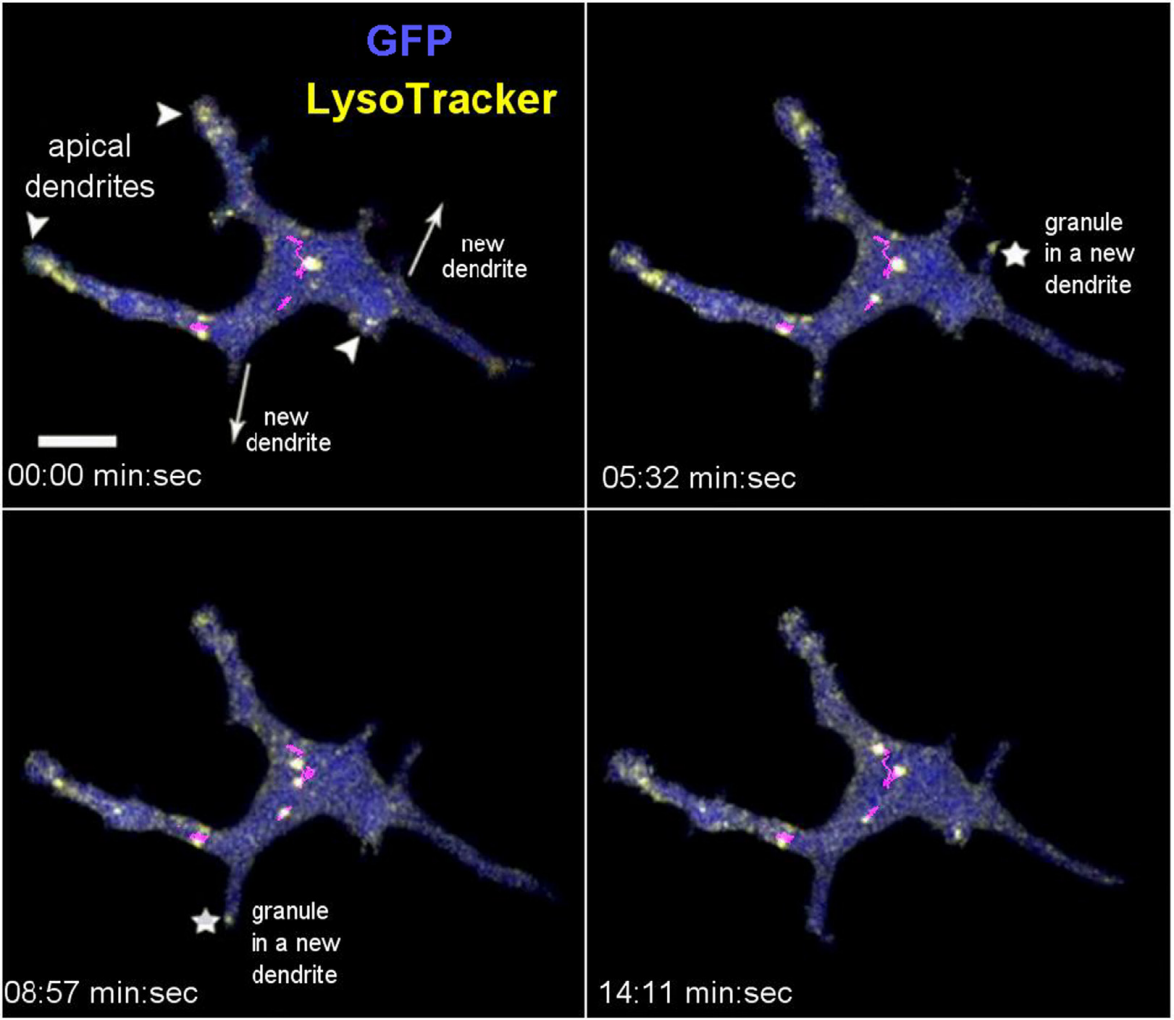
*In vivo* dynamics of dendritic epidermal T cell granules labeled with LysoTracker. The panel shows maximum intensity projections from a video sequence. The arrowheads point to the apical dendrites (based on 3-D inspection), the arrows indicate the direction of new dendrite growths, and the asterisks indicate the accumulating granules. The magenta lines represent tracks of several mid-body granules (see Movie S1 in Supplementary Material). Scale bar = 5 µm.

Having established three methods for intravital labeling of DETC granules (i.e., using Vγ5 antibody, CTB, or LT), we proceeded to characterize the steady state granule dynamics. The LT labeling method was rapid and not expected to interfere with any cellular processes, but could not exclude the possibility of the dye leakage between cells. The Vγ5 and CTB labeling required a 6-day rest, but excluded fluorescence leakage and highlighted a somewhat different range of granules. Intravital mouse time-lapse microscopy of LT-stained granules revealed highly dynamic behavior, the lysosomes seemingly fusing with each other and splitting (Figure 3; Movies S1 and S2 in Supplementary Material). The median instantaneous velocity was 0.84 µm/min (0.35–2.04 µm/min—25–75% percentile, respectively), while the fastest vesicles were typically small (FWHM ≤ 0.3 µm) and moved at 8.5 µm/min. The maximum instantaneous speed of the largest lysosomes was within the range of 1.1–2.4 µm/min and their short-range motions seemed to be random over the relatively short observation time. On occasion, we observed the process of a new dendrite forming by localized budding off followed by back and forth length changes ending with the distal end anchoring, presumably through a PALP. During this process, which took several to tenths of minutes, lysosomes entered into the new dendrite soon after the budding off and then accumulated at the end after the length of dendrite has stabilized (Figure 3; Movies S1 and S2 in Supplementary Material). These observations suggested that the accumulations of lysosomes in DETC’s apical dendrite endings was a result of granule transport.

### Dynamics of CTB-Labeled Granules and Comparison With Non-DETC

**Figure 4:**
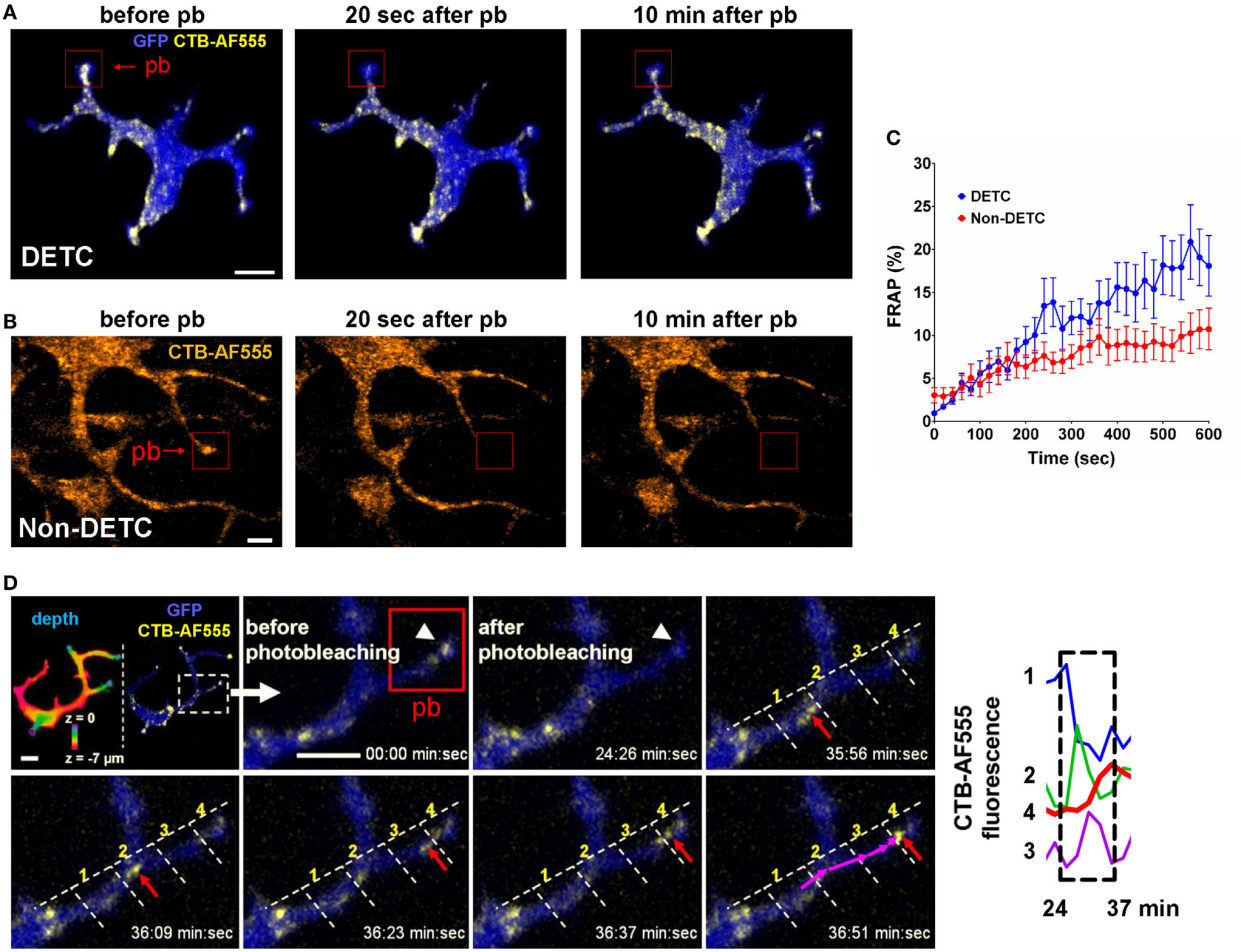
*In vivo* dynamics of dendritic epidermal T cell (DETC) granules labeled with CTB-AF555 in IL2p8-GFP mouse ear. **(A)** Fluorescence recovery after photobleaching (FRAP) in a DETC dendrite end. The red rectangle area was photobleached (pb) and the cell was imaged for 10 min (see Movie S3 in Supplementary Material). **(B)** FRAP in a dermal non-DETC dendrite end. The red rectangle area was photobleached and the cell was imaged for 10 min (see Movie S4 in Supplementary Material). **(C)** Cumulative analysis of FRAP kinetics for multiple dendrite ends (*n* = 15 cells in five mice). Each point is a mean ± SEM. **(D)** Example of cargo movement along dendrite length. Upper left: color-coded depth—the apical localizations are blue-green and basal localizations are red. The dashed rectangle indicates zoom area. AF555 was photobleached in the red rectangle area. AF555 signal was measured in the four regions of interest (ROI) along the dendrite. The red arrow points to granule movement. The graph shows a rapid transition of AF555 (granule) intensity through the four ROIs, this movement starting after 24 min. Scale bars = 5 µm.

We used steady state CTB and Vγ5 labeling to evaluate the dynamics of intracellular cargo transport by measuring the kinetics of intravital FRAP. Lack of intercellular fluorophore diffusion was ascertained by the lack of signal recovery upon whole-cell FRAP (Figure S4 in Supplementary Material). Using CTB, we compared DETCs to the CTB^+^ non-DETC cells in the dermis. Figure 4A and Movie S3 in Supplementary Material show an example of FRAP experiment whereby the photobleaching was localized in a DETC apical dendrite end characteristic of a PALP, and Figure 4B and Movie S4 in Supplementary Material show a similar experiment on a dendrite of a dermal non-DETC CTB^+^ cell. In DETCs, fluorescence re-emerged in the photobleached dendrite with the recovery half-life estimated for 10–45 min (Figure 4C, blue curve). By contrast, similar distance and shape FRAP kinetics in non-DETC dendrites were about twice slower (Figure 4C, red curve), also in comparison to non-DETC mid-bodies (Figure S5 in Supplementary Material). Although granule transport in cell bodies is not unexpected *per se*, this comparison showed that the capacity of DETC for dendrite-guided cargo transport was more profound than in non-DETCs.

Time-lapse recordings of steady state CTB granules in DETCs revealed individual granule motilities. The median instantaneous velocity was 1.22 µm/min (0.51–2.50 µm/min—25–75% percentile, respectively). CTB granules moved from the mid-body along the lengths of dendrites in jumping fashion and the fluorescence accumulated at the apical end (Figure 4D). In this example, fluorescence intensity spiked sequentially through the regions 1–4 as the vesicle moved through the branched dendrite from 24 to 37 min. These recordings demonstrated that CTB-labeled vesicles were transported from the mid-body through dendrites toward dendrite ends.

### Dynamics of TCR-Labeled Granules and Retrograde Transport

**Figure 5:**
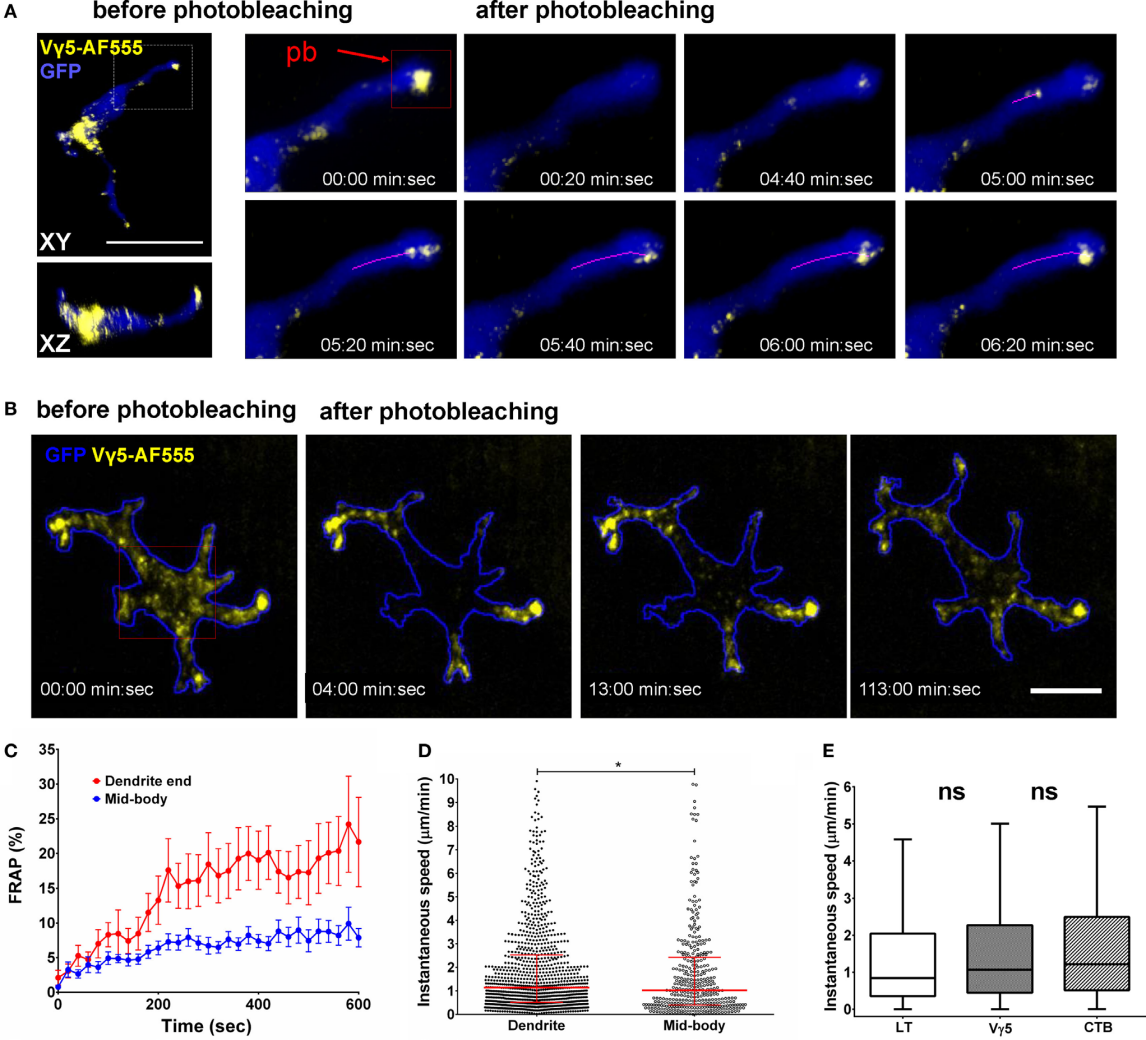
*In vivo* dynamics of dendritic epidermal T cell (DETC) granules labeled by intradermal anti-Vγ5-AF555. **(A)** Fluorescence recovery after photobleaching (FRAP) in an apical dendrite end. AF555 fluorescence was photobleached in the red rectangle area centered on the end of an apical-facing dendrite (pb) and the entire cell was imaged, in 3-D, every 20 s for 10 min. The magenta line represents a track of moving granule. **(B)** FRAP in DETC mid-body. The photobleaching was located in the red rectangle area. Cell outline based on GFP signal. **(C)** FRAP analysis, *n* = 15 cells in five mice. **(D)** Comparison of instantaneous granule velocities in DETC dendrites vs. mid-bodies. Each dot is the speed of a single vesicle, data pooled from *n* = 15 cells in five mice. The horizontal line and whiskers are the median and interquartile ranges. **(E)** Comparison of instantaneous granule velocities depending on labeling method. Data pooled from *n* = 15 cells in five mice, shown as box with median and interquartile range with Tukey whiskers. Scale bars = 10 µm.

Finally, we evaluated the steady state dynamics of DETC granules that were labeled by anti-Vγ5-TCR antibody internalization. Given that CTB-based labeling could be associated with a degree of inflammatory activation, Vγ5 labeling should be free from such effects. As for CTB, we waited for 6 days from the intradermal injection to allow for complete internalization and washout and for the skin to return to a steady state. After photobleaching the apical dendrite endings, we observed movement of vesicles from DETC bodies toward the photobleached dendrites within 6 min (Movies S5 and S6 in Supplementary Material). Figure 5A depicts the movement of individual vesicles that are arriving at a photobleached dendrite end. Based on multiple FRAP experiments, the median instantaneous velocity of granule movement was 1.07 µm/min (0.44–2.27 µm/min—25–75% percentile, respectively) and maximum instantaneous velocity was 4.23 µm/min (1.75–7.66 µm/min—25–75% percentile, respectively). As for LT labeling, we observed that newly formed dendrites were quickly filled with Vγ5-labeled vesicles migrating into the emergent tips (Movie S7 in Supplementary Material).

To test for the presence of retrograde granule movement, i.e., from dendrite ends toward the mid-bodies, we photobleached mid-cell body areas such that the only remaining fluorescence was located in dendrite endings (Figure 5B; Movies S8 and S9 in Supplementary Material). In this setting, we observed fluorescence entering the mid-body regions from dendrite ends. However, the retrograde transport kinetics was about half of the anterograde movements and the extrapolated FRAP half time was approximately 80 min (Figure 5C). Based on individual Vγ5 granule tracking in multiple cells, the instantaneous velocities of the granules were somewhat higher in dendrites compared to mid-bodies (Figure 5D). The instantaneous velocities of CTB, TCR, and LT granules were not statistically different (Figure 5E).

## Discussion

This study follows upon the discovery of DETC *in vivo* trans-epidermal polarization through the steady state TCR activation in the PALPs (5). The apical-polarized dendritic morphology of DETCs is a puzzling feature of this T cell lineage, reminiscent of their epidermal dendritic cohabitants, Langerhans cells. It is thought to facilitate a remote probing of the surrounding microenvironment including the barrier-forming apical squamous keratinocyte layers while maintaining cell body residence at a safe distance (13). A distinctive feature of DETCs, the PALPs contain distinct accumulations of cytoplasmic granules containing varying amounts of TCR, GM1, and LAMP-1 (5). In this report, we focused on the intracellular dynamics of DETC granules in the physiological microenvironment of intact skin at steady state. Our results demonstrate that DETC’s long cellular projections sustain trans-epidermal trafficking of intracellular cargo, both in anterograde and retrograde fashion, i.e., toward and away from the PALPs. This way, DETCs can be considered a conduit system for cargo transport across the epidermis. To the best of our knowledge, this aspect of intraepithelial γδ T cell biology, i.e., *in vivo* intracellular granule transport, has not been studied before in DETCs or other intraepithelial T cell systems. The comparison of FRAP kinetics in DETC dendrites with that in nearby comparable cellular protrusions of dermal cells showed that the rate of transport in DETCs is higher. We could not compare DETCs to Langerhans cells because we did not find a common scheme for granule labeling in these cells other than with LT, which is not suitable for FRAP.

One tantalizing question that remains to be addressed is about the exact functional biological purpose of the apical dendrite granule accumulations, which has to be considered in the context of the PALPs. The presence of LT and mature lysosome/exocytosis marker LAMP-1 in DETC apical granules that also colocalized with Vγ5 and/or CTB fluorescence (Figure 2) suggests that some of the granules represent mature lysosomes, which could be poised for externalization, and that TCR and GM1/CTB internalization pathways ultimately feed into mature lysosomes. The latter notion and the relatively lower CTB/LT co-localization in mid-bodies compared to dendrites, where most LT fluorescence accumulated, is consistent with the process of membrane GM1 recycling by lysosomal sorting (14). This and other dissimilarities between the intracellular fates and steady state localizations of Vγ5 antibody and CTB fluorescence likely reflect expected variances in ligand and label trafficking and degradation. Considering that DETCs produce granzymes and perforin and can kill target cells by cytotoxic/LAMP-1 granule release (6–8, 15), and that cytolytic granules are a subset of lysosomes, one exciting proposition is that some of the PALP’s lysosomal and LAMP-1 granules that accumulate in PALPs contain cytotoxic factors. The presence of lysosomal and TCR-containing granules just underneath the sites of DETC TCR steady state activation, i.e., the PALPs (5) and now the presence of steady state transport for dendrite end granule accumulation and retrograde transport resemble the immunological synapses that form, albeit transiently, between antigen-specific cytotoxic T lymphocytes and their cellular targets (5, 16, 17). Another resemblance is with NK cell’s remote synapse-like structures that form at the tips of “membrane nanotubes” and appear to support target cell killing (18). Other possible functions of DETC granule transport to the apical epidermis could be to facilitate targeted secretion of keratinocyte differentiation or repair factors. By demonstrating three methods for intravital DETC granule labeling, and by revealing the pattern of granule dynamics in otherwise un-manipulated/steady state, the current study should facilitate further functional and mechanistic investigations. An interesting aspect of any biological function of dendrite-guided DETC cargo transport is that it will be lost when DETCs are activated and round up.

Our demonstration of the existence of granule transport in DETC dendrites was enabled by the intravital granule labeling and tracking and by FRAP technique. The use of LT required independent labeling of DETCs with cytoplasmic fluorescent protein reporter (IL2p8-GFP) for digital 3-D gating, but it was convenient, rapid, and practically non-disturbing. LT labeling was suitable for granule tracking but less so for FRAP because the small molecule dye could, in principle, diffuse between granules or cells rather freely. Another limitation was that LT-based granule tracking was strictly limited to the inside of DETC bodies due to the need for image digital gating. By contrast, Vγ5 and CTB-based intravital labeling was not expected to allow for fluorescence diffusion (although carrier protein degradation would eventually ensue) thereby enabling FRAP experimentation. These methods should be useful to follow granules fates outside DETC bodies. One disadvantage of these labeling schemes was the possibility of transient cellular responses to the ligands hence the need for several days of steady state re-establishment. As mentioned earlier, anti-DETC TCR antibody did not deplete these cells *in vivo*—of note for experiments where DETC depletion might be desired.

The magnitude of CTB selectivity for DETCs among other cells in the epidermis was quite remarkable, suggesting that DETCs represent a major source (or recipient) of GM1 ganglioside in epidermis. While DETC-bound Vγ5 antibody was internalized relatively slowly, over more than 24 h, CTB internalization occurred much faster, within one and a half hour. This could reflect the underlying dynamics of GM1 ganglioside or CTB pentameric structure hence stronger avidity and capacity for cross-linking. Alternatively, the rapid labeling of DETC granules by CTB internalization could be related to the previously described process of physiological transport of gangliosides from the plasma membrane to intralysosomal membranes in cultured fibroblasts (14). If secreted, GM1 production by DETCs could be of local consequence for keratinocyte differentiation (19).

The dynamic imaging here confirmed our prior findings of the apical dendrite-terminal lysosome accumulation and it demonstrated that these lysosomal clusters are relatively long-lived and confined, as opposed to diffusing randomly in and out of the end-terminal dendrite swellings. Interestingly, these experiments revealed highly dynamic movement of lysosomal granules within the dendrites and cell bodies, including directional inflow into newly formed dendrites. This anterograde transport was reminiscent of “long processive runs,” as if it followed tracks, described by Rodionov et al. (20), likely driven by molecular motors and cytoskeletal structures such as microtubules. In this respect, an interesting puzzle arises when one considers the known role of the microtubule-organizing center (MTOC) in the movement of cargo at the immunological synapse of classical αβ T cells. Therefore, the directional movement of granule subsets is associated with the MTOC relocalization toward the synapse (21, 22). This mechanism clearly cannot operate in DETCs at steady state because of the multiplicity of the PALPs.

Given that intradermal anti-Vγ5-TCR antibody (or CTB) did not cause perceptible morphological changes that would indicate acute cellular activation, such as cell rounding up, and consistent with the presence of non-phosphorylated TCR in submerged clusters underneath the PALPs surface (5), our results demonstrate that DETC TCR can be readily internalized without perturbing the cell behavior in a gross manner. Considering the ultimate trafficking of Vγ5 antibody fluorescence into lysosomal and LAMP-1 granules including in the PALPs, and knowing that TCRs of classical αβ T cells are continuously internalized and recycled back to the cell surface (23, 24), it is possible that the physiological accumulation of TCR in the PALPs is in part maintained by endosome-mediated TCR recycling. However, we could not yet establish if the PALPs are the sites of physiological TCR internalization. DETCs could use TCR and/or other cell membrane component internalization to efficiently probe their extracellular surroundings. In this respect, it would be interesting to know whether steady state and/or acute TCR binding to endogenous ligands, which remain to be discovered, would be associated with TCR internalization similar to that by anti-Vγ5 antibody, and weather the endogenous ligands would remain bound to the TCR and co-internalized. The retrograde transport could serve multiple purposes such as to simply retrieve molecules for disposal or recycling, or to probe the molecular composition of epidermal barrier for communication into the dermis. The uncovering of DETC’s dendrite-mediated cargo transport opens a host of new directions for further functional studies.

## Ethics Statement

All animal manipulations were approved by the Institutional Animal Care and Use Committee, The University of Texas MD Anderson Cancer Center, Houston, TX, USA, or the Local Ethics Committee for Experiments on Animals at the Institute of Immunology and Experimental Therapy, Wroclaw, Poland.

## Author Contributions

GC and TZ designed the studies, analyzed and interpreted the results. GC, MT, and MZ obtained and analyzed the data. GC, MT, and TZ wrote the manuscript.

## Conflict of Interest Statement

The authors declare that the research was conducted in the absence of any commercial or financial relationships that could be construed as a potential conflict of interest.

## Supplementary Material

Figure S1 Internalization of Vγ5-AF647 and CTB-AF555 in dendritic epidermal T cells (DETCs). Images show examples of DETCs after 10 min, 90 min, or 24 h from intradermal co-injection of CTB-AF555 and anti-Vγ5-AF647. The white arrows point to the apical dendrites (based on 3-D z-stack inspection). Scale bar = 10 μm.

Figure S2 Vγ5 labeling is specific for dendritic epidermal T cells (DETCs). **(A)** Accumulation of anti-Vγ5-AF555 antibody in DETCs but not in Langerhans (LC) or other cells. Left panel: dual reporter IL2p8-GFP (bright) and CD11c-YFP (dim) mouse skin imaged in the same channel. Middle panel: DETC and LC identification. **(B)** Comparison of anti-Vγ5-AF555 antibody (upper panel) with the isotype control (lower panel). Fluorescence intensity profiles are drawn along dashed regions to relate AF555 signals to GFP-labeled DETC bodies. Scale bar = 10 μm.

Figure S3 Identification of CTB-labeled granules. Immunofluorescence on skin samples 6 days after injection of fluorescent CTB. Steady state CTB-labeled granules colocalize with LAMP-1. The white arrows point to apical dendrite endings, determined by 3-D z-stack inspection.

Figure S4 Whole-cell fluorescence recovery after photobleaching (FRAP). The lack of fluorescence recovery in the whole-cell photobleached areas demonstrates that CTB **(A)** and Vγ5 **(B)** labeling did not diffuse from the intercellular spaces or by leaking from the neighboring cells. Fluorescence intensity profiles are based on AF555 signal measured in the whole cell.

Figure S5 Mid-body fluorescence recovery after photobleaching (FRAP) in dermal non-dendritic epidermal T cells (DETCs) labeled by CTB-AF555 (related to Figure 4B). Ear skin of a mouse was labeled with intradermal CTB-AF555. Laser scanning microscope was focused in the dermis and the area in the red rectangle was photobleached followed by time-lapse imaging of the FRAP. Lower panel: FRAP quantification for dendrite ends and mid-bodies for multiple FRAP experiments on 10–13 cells in two mice each. Images in “orange hot” intensity color palette. Scale bar = 10 μm.

Movie S1 Dynamics of lysosomes in a dendritic epidermal T cell (DETC) *in vivo*. Imaging was performed 1 h after intradermal injection of LysoTracker (LT) Red into a healthy IL2p8-GFP mouse. Depth color coding indicates apical layers in purple, also shown with the arrowheads. The arrows point to the direction of a new dendrite formation. The new dendrites are quickly populated by LT-stained granules. LT signal based on GFP gate.

Movie S2 Dynamics of lysosomes in a dendritic epidermal T cell (DETC) *in vivo*. Imaging was performed 1 h after intradermal injection of LysoTracker (LT) Red into a healthy IL2p8-GFP mouse. Four optical sections from a z-stack are shown with raw (non-GFP-gated) LT signal. Right top side: maximum intensity projection of the z-stack with LT signal based on GFP gate. Right bottom side: LT signal based on GFP gate in “fire” intensity palette.

Movie S3 *In vivo* dynamics of dendritic epidermal T cell (DETC) intracellular granules labeled by CTB-AF555 internalization. Imaging was performed 11 days after intradermal injection of CTB-AF555 into a healthy IL2p8-GFP mouse. Depth color coding indicates apical layers in purple, also shown with the arrowheads. The white squares indicate the areas subjected to photobleaching. The arrows point to newly arrived granules.

Movie S4 *In vivo* dynamics of intracellular granules labeled by CTB-AF555 internalization in dermal non-dendritic epidermal T cells (DETCs). The white squares indicate the areas (dendrite end or mid-body) subjected to photobleaching.

Movie S5 *In vivo* dynamics of dendritic epidermal T cell (DETC) intracellular granules labeled by internalized anti-Vγ5-AF647. Imaging was performed 6 days after the antibody intradermal injection into a healthy IL2p8-GFP mouse. Individual video frames represent temporal projections of 376 s sequences. The white rectangle indicates the area subjected to photobleaching.

Movie S6 *In vivo* dynamics of anti-Vγ5-T cell receptors (TCR)-AF555 labeled granules at dendritic epidermal T cell (DETC) dendrite ends. Imaging was performed 6 days after intradermal injection of anti-Vγ5-AF555 antibody into a healthy IL2p8-GFP mouse. The white rectangles indicate the areas subjected to photobleaching. GFP-based depth color coding indicates apical layers in blue. AF555 signal is represented in “fire” intensity palette. Movie is a combined recording of three cells.

Movie S7 *In vivo* dynamics of anti-T cell receptors (TCR)-AF647 labeled granules in new dendrites and in a dendritic epidermal T cell (DETC) mid-body area that is not connected to a dendrite. Imaging was performed 6 days after intradermal injection of anti-Vγ5 TCR-AF647 antibody into one ear of a healthy IL2p8-GFP mouse. Bottom panels show the AF647 channel intensity using the “fire” intensity palette. The white rectangle indicates the area subjected to photobleaching, also indicated with the white arrow. For this cell, the photobleached area is located over a part of cell mid-body that is not a dendrite, and not connected to a dendrite. The insets shown on the right side represent zoomed-in areas to point to the dendrite ends labeled with the corresponding numbers. The red arrows point to the areas of new dendrite appearance, which are quickly populated by fluorescent granules.

Movie S8 *In vivo* dynamics of anti-Vγ5-T cell receptors (TCR)-AF555 labeled granules at dendritic epidermal T cell (DETC) mid-body. Imaging was performed 6 days after intradermal injection of anti-Vγ5-AF555 antibody into a healthy IL2p8-GFP mouse. The white rectangles indicate the areas subjected to photobleaching. GFP-based depth color coding indicates apical layers in blue. AF555 signal is represented in “fire” intensity palette. Movie is a combined recording of three cells.

Movie S9 *In vivo* dynamics of anti-Vγ5-T cell receptors (TCR)-AF647 labeled granules at dendritic epidermal T cell (DETC) dendrite base. Imaging was performed 6 days after intradermal injection of anti-Vγ5-AF647 antibody into a healthy IL2p8-GFP mouse. The white rectangle indicates the area subjected to photobleaching.
