# Supplementary figures and images for: Epidermal T Cell Dendrites Serve as Conduits for Bidirectional Trafficking of Granular Cargo
Source: Front Immunol. 2018 Jun 22;9:1430. doi: 10.3389/fimmu.2018.01430 (PMC6023976; doi:10.3389/fimmu.2018.01430)

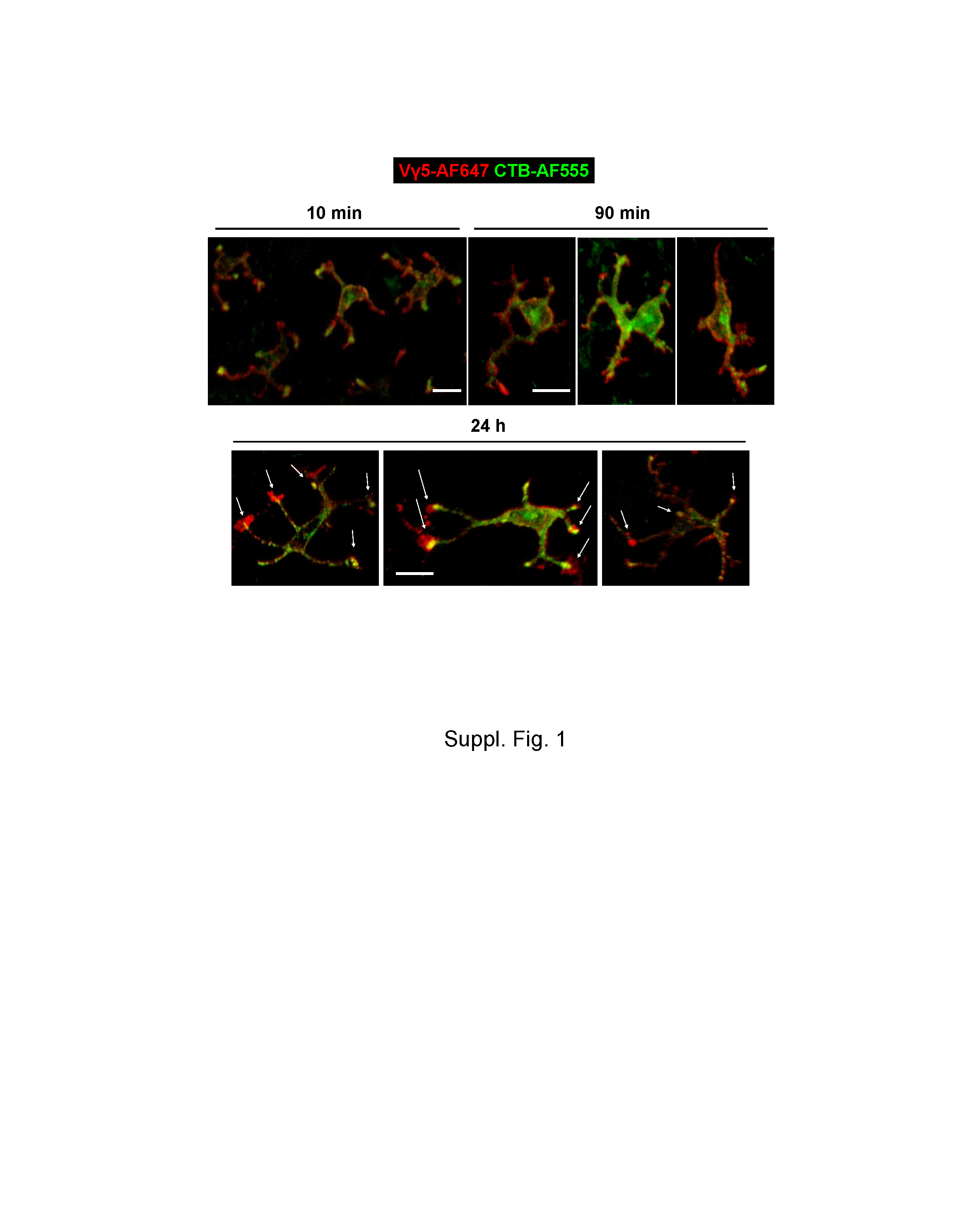

Supplement: Figure S1 — Internalization of Vγ5-AF647 and CTB-AF555 in dendritic epidermal T cells (DETCs). Images show examples of DETCs after 10 min, 90 min, or 24 h from intradermal co-injection of CTB-AF555 and anti-Vγ5-AF647. The white arrows point to the apical dendrites (based on 3-D z-stack inspection). Scale bar = 10 μm. [file image_1.tiff]

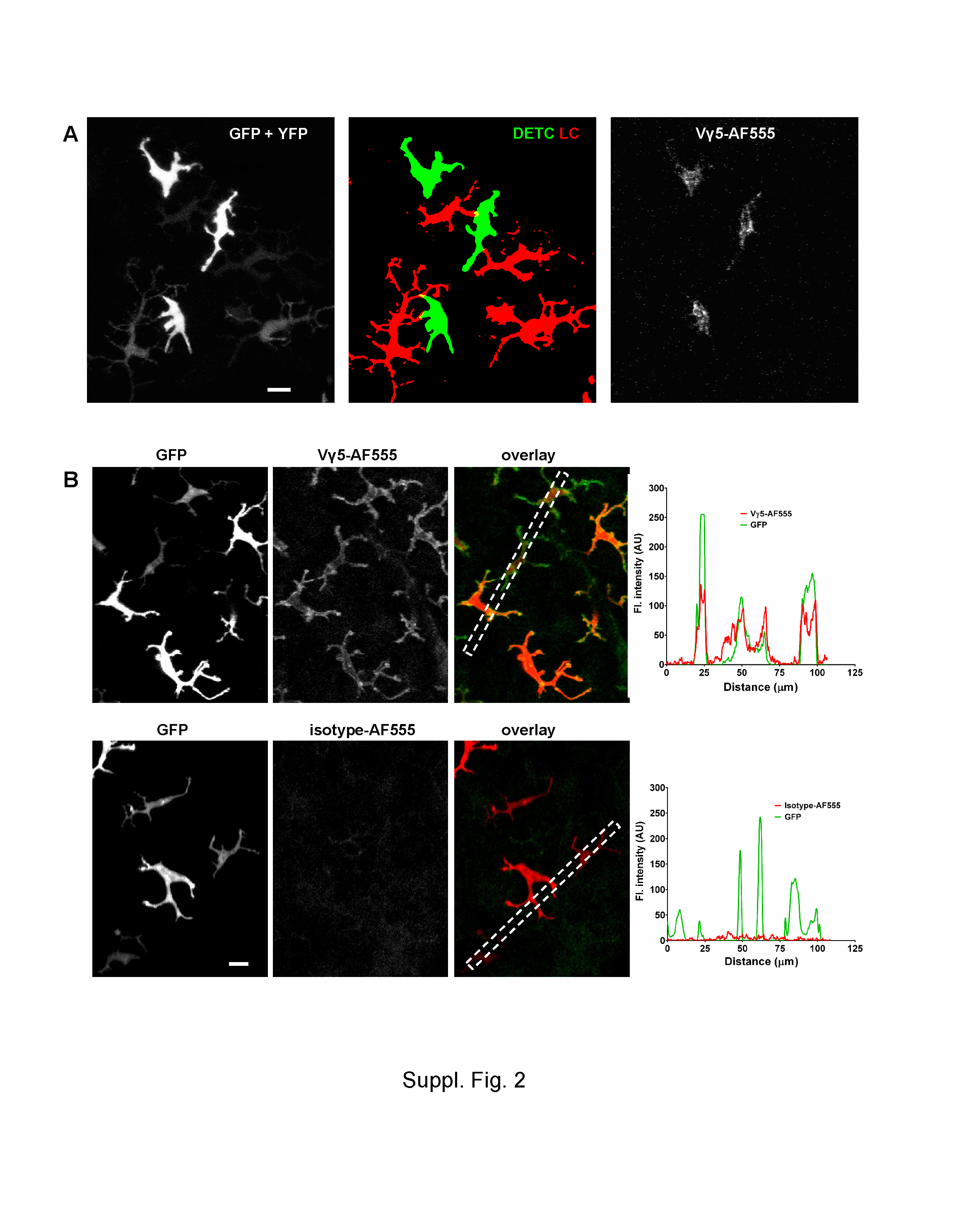

Supplement: Figure S2 — Vγ5 labeling is specific for dendritic epidermal T cells (DETCs). (A) Accumulation of anti-Vγ5-AF555 antibody in DETCs but not in Langerhans (LC) or other cells. Left panel: dual reporter IL2p8-GFP (bright) and CD11c-YFP (dim) mouse skin imaged in the same channel. Middle panel: DETC and LC identification. (B) Comparison of anti-Vγ5-AF555 antibody (upper panel) with the isotype control (lower panel). Fluorescence intensity profiles are drawn along dashed regions to relate AF555 signals to GFP-labeled DETC bodies. Scale bar = 10 μm. [file image_2.tiff]

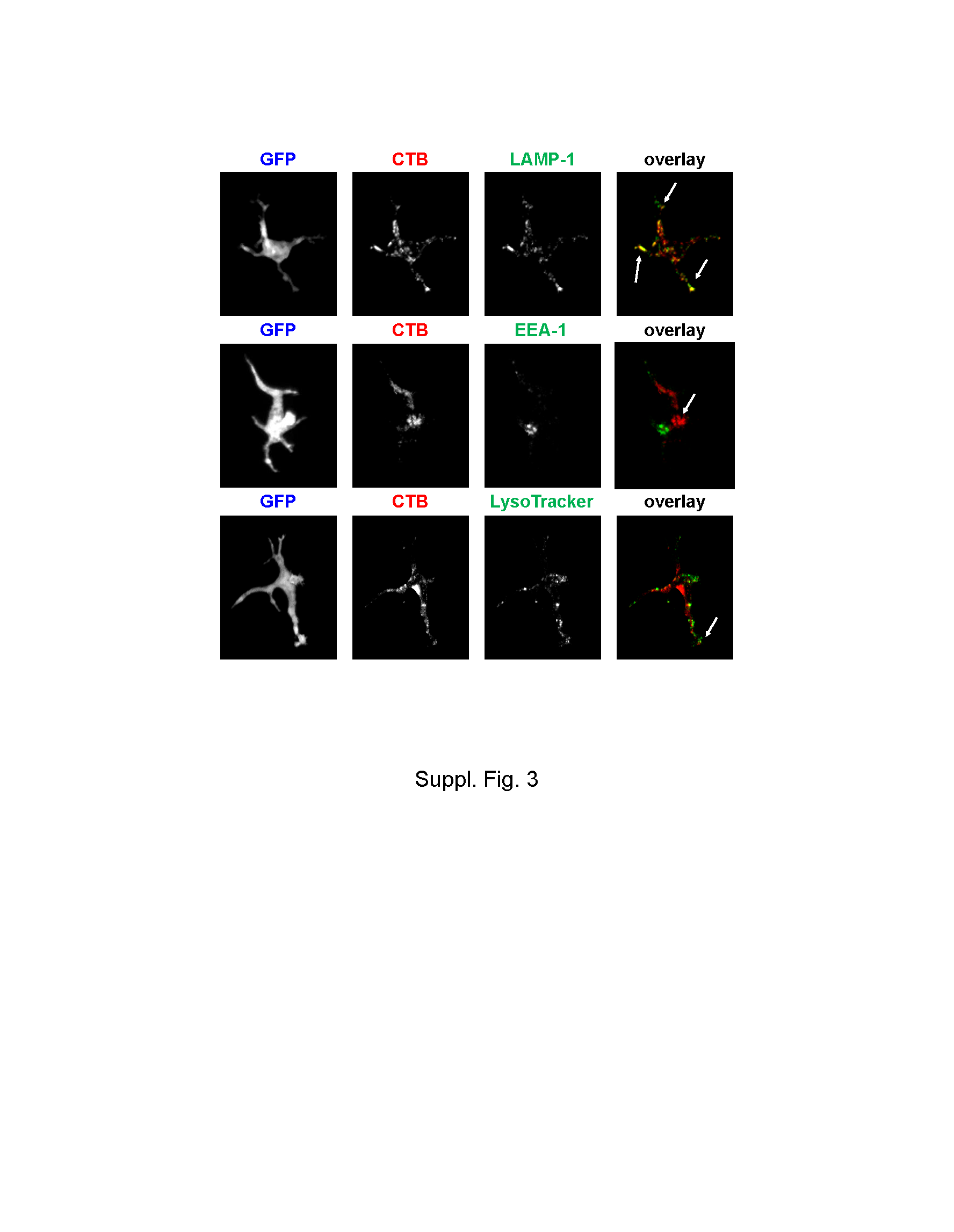

Supplement: Figure S3 — Identification of CTB-labeled granules. Immunofluorescence on skin samples 6 days after injection of fluorescent CTB. Steady state CTB-labeled granules colocalize with LAMP-1. The white arrows point to apical dendrite endings, determined by 3-D z-stack inspection. [file image_3.tiff]

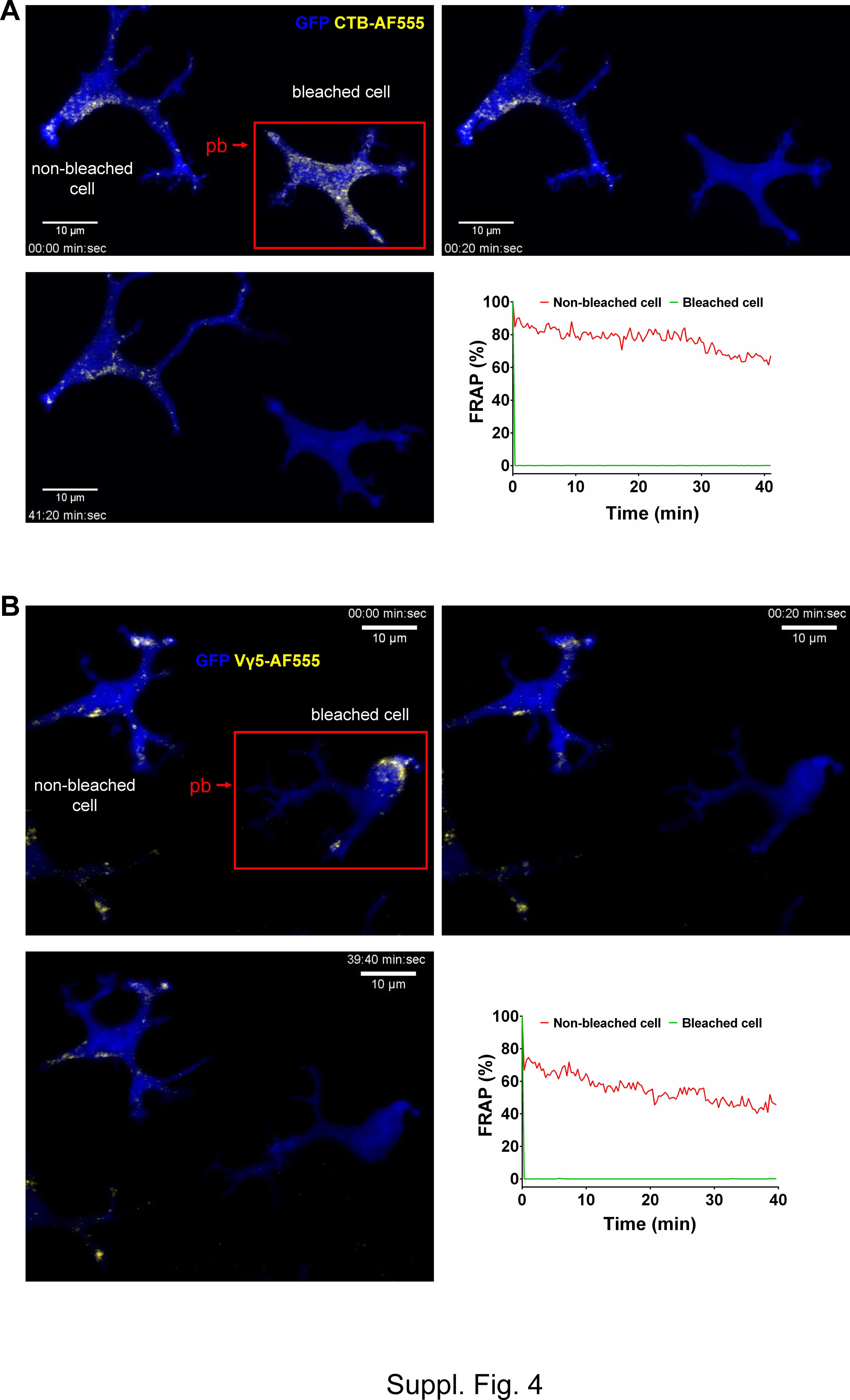

Supplement: Figure S4 — Whole-cell fluorescence recovery after photobleaching (FRAP). The lack of fluorescence recovery in the whole-cell photobleached areas demonstrates that CTB (A) and Vγ5 (B) labeling did not diffuse from the intercellular spaces or by leaking from the neighboring cells. Fluorescence intensity profiles are based on AF555 signal measured in the whole cell. [file image_4.tif]

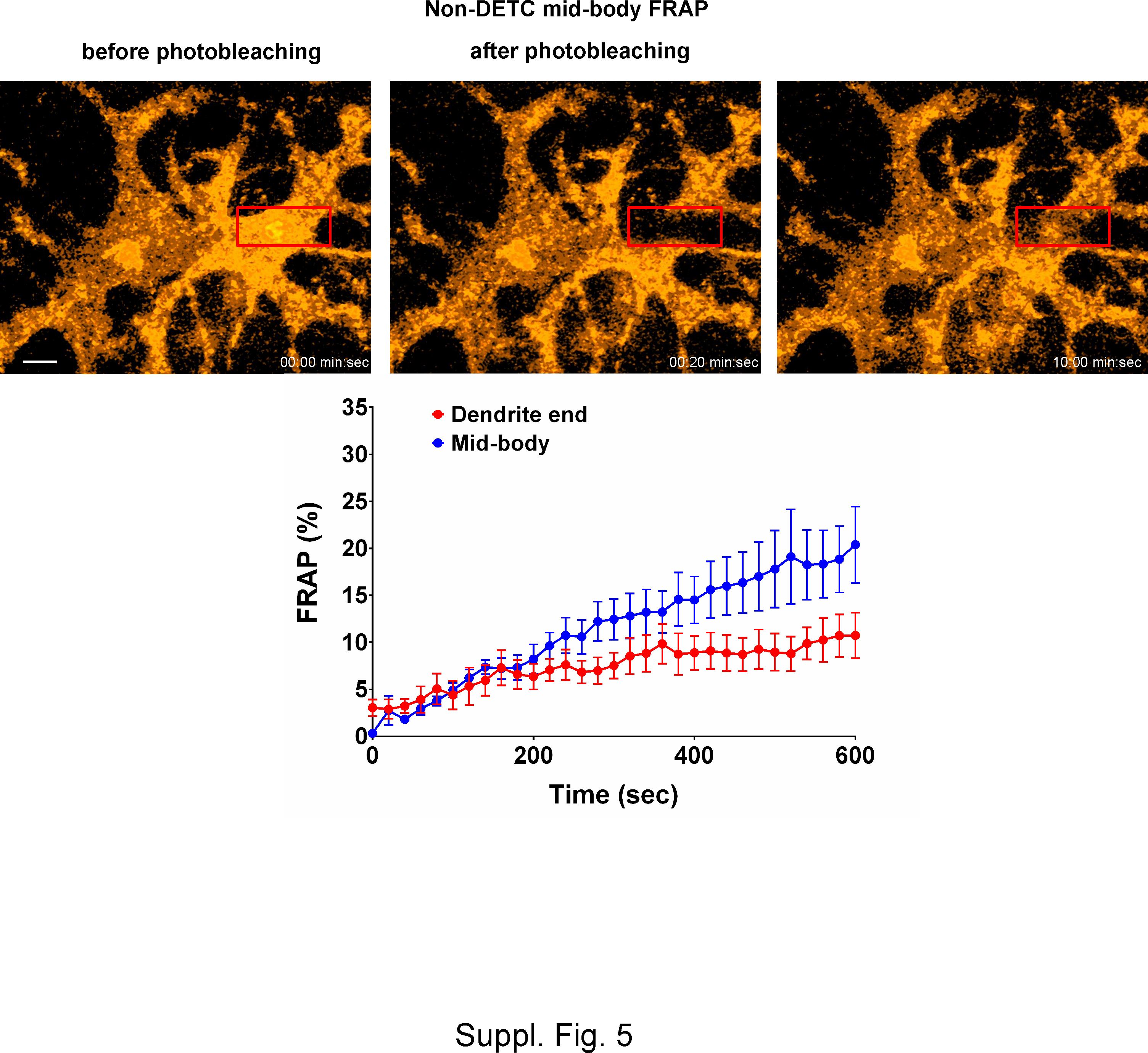

Supplement: Figure S5 — Mid-body fluorescence recovery after photobleaching (FRAP) in dermal non-dendritic epidermal T cells (DETCs) labeled by CTB-AF555 (related to Figure 4B). Ear skin of a mouse was labeled with intradermal CTB-AF555. Laser scanning microscope was focused in the dermis and the area in the red rectangle was photobleached followed by time-lapse imaging of the FRAP. Lower panel: FRAP quantification for dendrite ends and mid-bodies for multiple FRAP experiments on 10–13 cells in two mice each. Images in “orange hot” intensity color palette. Scale bar = 10 μm. [file image_5.tif]
